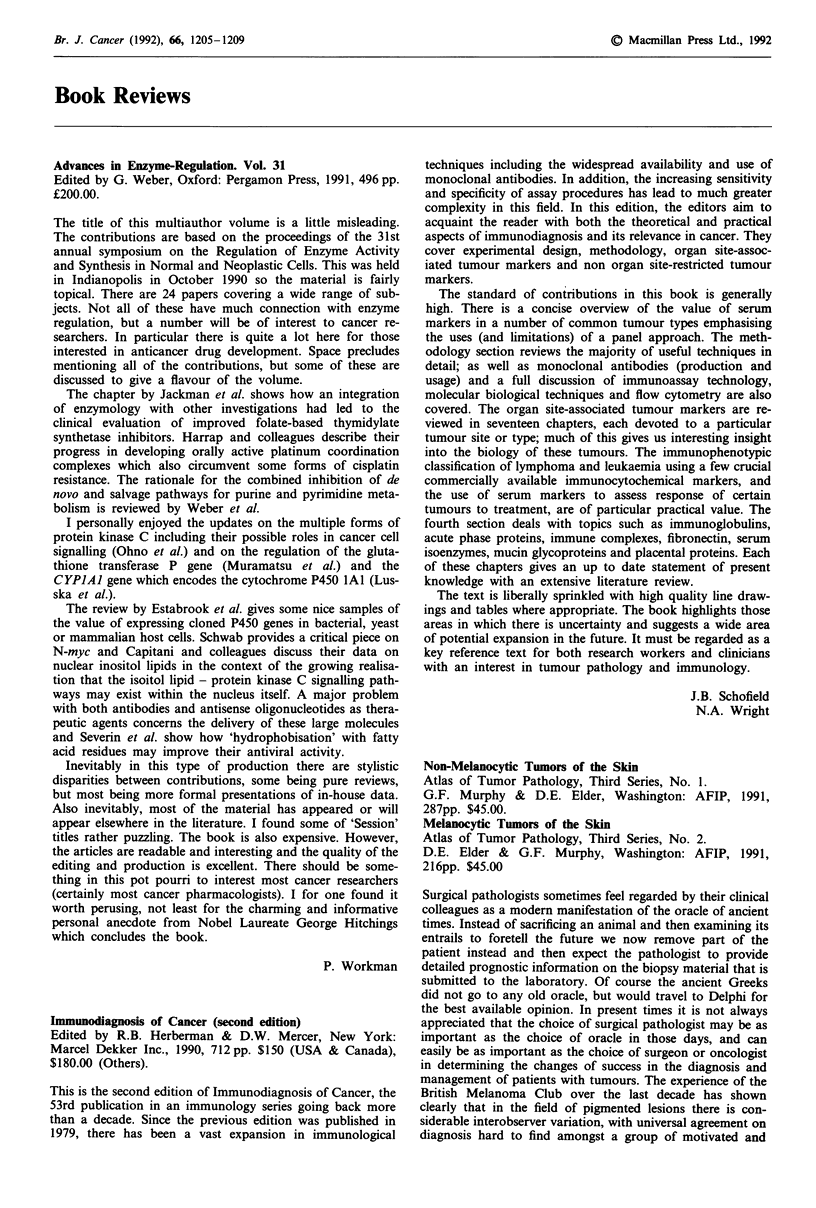# Advances in Enzyme-Regulation. Vol. 31

**Published:** 1992-12

**Authors:** P. Workman


### Book Reviews

**Advances in Enzyme-Regulation. Vol. 31**
Edited by G. Weber, Oxford: Pergamon Press, 1991, 496 pp. £200.00.

The title of this multiauthor volume is a little misleading. The contributions are based on the proceedings of the 31st annual symposium on the Regulation of Enzyme Activity and Synthesis in Normal and Neoplastic Cells. This was held in Indianopolis in October 1990 so the material is fairly topical. There are 24 papers covering a wide range of subjects. Not all of these have much connection with enzyme regulation, but a number will be of interest to cancer researchers. In particular there is quite a lot here for those interested in anticancer drug development. Space precludes mentioning all of the contributions, but some of these are discussed to give a flavour of the volume.

The chapter by Jackman *et al.* shows how an integration of enzymology with other investigations had led to the clinical evaluation of improved folate-based thymidylate synthetase inhibitors. Harrap and colleagues describe their progress in developing orally active platinum coordination complexes which also circumvent some forms of cisplatin resistance. The rationale for the combined inhibition of *de novo* and salvage pathways for purine and pyrimidine metabolism is reviewed by Weber *et al.*

I personally enjoyed the updates on the multiple forms of protein kinase C including their possible roles in cancer cell signalling (Ohno *et al.*) and on the regulation of the glutathione transferase P gene (Muramatsu *et al.*) and the *CYP1A1* gene which encodes the cytochrome P450 1A1 (Lusska *et al.*).

The review by Estabrook *et al.* gives some nice samples of the value of expressing cloned P450 genes in bacterial, yeast or mammalian host cells. Schwab provides a critical piece on N-*myc* and Capitani and colleagues discuss their data on nuclear inositol lipids in the context of the growing realisation that the isoitol lipid – protein kinase C signalling pathways may exist within the nucleus itself. A major problem with both antibodies and antisense oligonucleotides as therapeutic agents concerns the delivery of these large molecules and Severin *et al.* show how 'hydrophobisation' with fatty acid residues may improve their antiviral activity.

Inevitably in this type of production there are stylistic disparities between contributions, some being pure reviews, but most being more formal presentations of in-house data. Also inevitably, most of the material has appeared or will appear elsewhere in the literature. I found some of 'Session' titles rather puzzling. The book is also expensive. However, the articles are readable and interesting and the quality of the editing and production is excellent. There should be something in this pot pourri to interest most cancer researchers (certainly most cancer pharmacologists). I for one found it worth perusing, not least for the charming and informative personal anecdote from Nobel Laureate George Hitchings which concludes the book.

P. Workman